# Occupational Exposure to Swine, Poultry, and Cattle and Antibody Biomarkers of *Campylobacter jejuni* Exposure and Autoimmune Peripheral Neuropathy

**DOI:** 10.1371/journal.pone.0143587

**Published:** 2015-12-04

**Authors:** Leora Vegosen, Patrick N. Breysse, Jacqueline Agnew, Gregory C. Gray, Irving Nachamkin, Kazim Sheikh, Freya Kamel, Ellen Silbergeld

**Affiliations:** 1 Department of Environmental Health Sciences, Johns Hopkins Bloomberg School of Public Health, Baltimore, MD, United States of America; 2 Division of Infectious Diseases, Duke University School of Medicine, Durham, NC, United States of America; 3 Department of Pathology and Laboratory Medicine, Perelman School of Medicine, University of Pennsylvania, Philadelphia, PA, United States of America; 4 Department of Neurology, University of Texas Medical School, Houston, TX, United States of America; 5 Epidemiology Branch, National Institute of Environmental Health Sciences (NIEHS), National Institutes of Health (NIH), Research Triangle Park, NC, United States of America; Cornell University, UNITED STATES

## Abstract

**Introduction:**

Foodborne *Campylobacter jejuni* infection has been associated with an increased risk of autoimmune peripheral neuropathy, but risks of occupational exposure to *C*. *jejuni* have received less attention. This study compared anti-*C*. *jejuni* IgA, IgG, and IgM antibody levels, as well as the likelihood of testing positive for any of five anti-ganglioside autoantibodies, between animal farmers and non-farmers. Anti-*C*. *jejuni* antibody levels were also compared between farmers with different animal herd or flock sizes. The relationship between anti-*C*. *jejuni* antibody levels and detection of anti-ganglioside autoantibodies was also assessed.

**Methods:**

Serum samples from 129 Agricultural Health Study swine farmers (some of whom also worked with other animals) and 46 non-farmers, all from Iowa, were analyzed for anti-*C*. *jejuni* antibodies and anti-ganglioside autoantibodies using ELISA. Information on animal exposures was assessed using questionnaire data. Anti-*C*. *jejuni* antibody levels were compared using Mann-Whitney tests and linear regression on log-transformed outcomes. Fisher’s Exact Tests and logistic regression were used to compare likelihood of positivity for anti-ganglioside autoantibodies.

**Results:**

Farmers had significantly higher levels of anti-*C*. *jejuni* IgA (p < 0.0001) and IgG (p = 0.02) antibodies compared to non-farmers. There was no consistent pattern of anti-*C*. *jejuni* antibody levels based on animal herd or flock size. A higher percentage of farmers (21%) tested positive for anti-ganglioside autoantibodies compared to non-farmers (9%), but this difference was not statistically significant (p = 0.11). There was no significant association between anti-*C*. *jejuni* antibody levels and anti-ganglioside autoantibodies.

**Conclusions:**

The findings provide evidence that farmers who work with animals may be at increased risk of exposure to *C*. *jejuni*. Future research should include longitudinal studies of exposures and outcomes, as well as studies of interventions to reduce exposure. Policies to reduce occupational exposure to *C*. *jejuni* should be considered.

## Introduction

Farmers and others who work closely with animals may be at elevated risk of exposure to several zoonotic pathogens including viruses and bacteria [1–8]. The pathogen *Campylobacter jejuni* is an avian commensal bacterium frequently carried by domesticated poultry and also carried by cattle and swine [9]. This zoonotic pathogen is of particular concern for human health because in addition to causing acute gastrointestinal illness, *C*. *jejuni* is also associated with post-infection sequelae. *C*. *jejuni* infection is the most commonly identified antecedent to Guillain-Barré Syndrome (GBS), an autoimmune peripheral neuropathy that is the leading cause of acute flaccid paralysis globally and in the U.S. [10–12]. The Centers for Disease Control and Prevention (CDC) estimates that foodborne *Campylobacter* spp. are associated with 845,024 illnesses, 8,463 hospitalizations, and 76 deaths in the U.S. per year [13].

*C*. *jejuni* is recognized as an important foodborne pathogen and thus may affect the general population. However, occupational exposures to farm animals at all stages of food production may also be an important source of *Campylobacter* infection [14]. Case-control studies have found significant positive associations between exposure to farm animals and *Campylobacter* infection [15,16]. A meta-analysis found that direct contact with farm animals was associated with an increased odds of *Campylobacter* infection [17]. Furthermore, elevated levels of anti-*C*. *jejuni* antibodies in poultry and meat processing workers were reported as early as 1981[18], as well as more recently [19]. Despite the evidence of occupational exposure to *C*. *jejuni*, little attention has been paid to the potential role of this exposure in inflammatory peripheral neuropathy.

We previously reported that poultry, swine, and cattle farmers in the Agricultural Health Study (AHS) had a higher prevalence of self-reported symptoms of peripheral neuropathy (numbness in hands or feet and weakness in arms or legs) compared to AHS farmers who did not work with animals [6,7]. These symptoms are not specific to autoimmune peripheral neuropathy and may be due to other causes including physical trauma. Therefore, the present study uses anti-*C*. *jejuni* antibodies as biomarkers of exposure and antiganglioside autoantibodies as biomarkers of autoimmune outcome.

**Fig 1:**
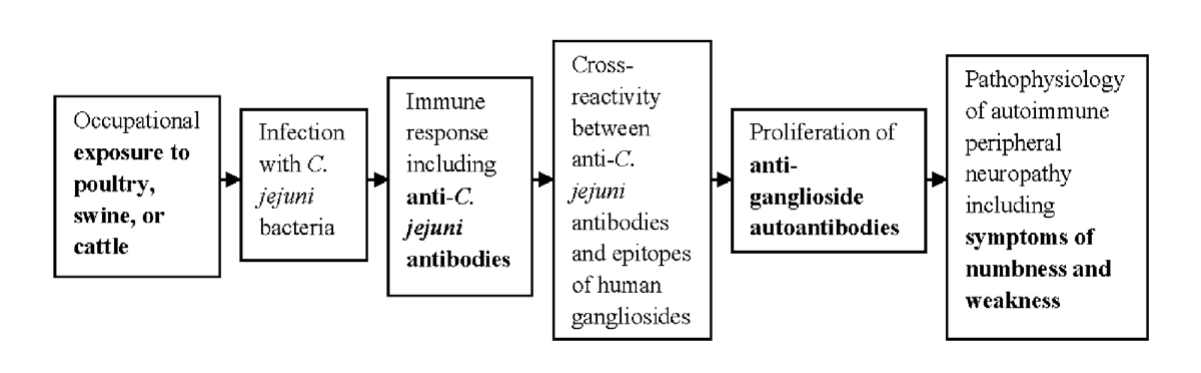
Schematic Depiction of Hypothesized Causal Pathway Between Occupational Exposure to Poultry, Swine, or Cattle and Development of Autoimmune Peripheral Neuropathy. Farmers and others who work with animals may be occupationally exposed to the avian commensal bacterium *Campylobacter jejuni*, which may result in infection and immune response. Molecular mimicry, or similarity in structure, between lipo-oligosaccharides (LOS) of *C*. *jejuni* bacteria and epitopes of human gangliosides may lead to the proliferation of anti-ganglioside autoantibodies and subsequent symptoms of autoimmune peripheral neuropathy.

The mechanism by which *C*. *jejuni* exposure leads to GBS and other inflammatory neuropathies is thought to involve molecular mimicry-associated autoimmunity, in which similarity in molecular structure between an immune-reactive epitope of a pathogen and a component of human tissue (self-epitope) leads to immune cross-reactivity with self-antigens [20–22]. The hypothesized pathway, involving molecular mimicry, between exposure to *C*. *jejuni* and the development of autoimmune peripheral neuropathy is illustrated in Fig 1.

Evidence indicates that structural similarities between lipo-oligosaccharides on the surface of *C*. *jejuni* and epitopes of human gangliosides are associated with autoantibodies directed against several gangliosides expressed in the nervous system including GM1, GD1a, GD1b, GQ1b, SGPG, GT1a, GD3, GM2, GD2, GA1, GM1b, GalNAc-GM1b, and GalNAc-GD1a [22,23]. Anti-ganglioside autoantibodies have been detected in serum from patients with autoimmune peripheral neuropathy. Different anti-ganglioside autoantibodies have been associated with different phenotypes of autoimmune peripheral neuropathy [24,25]. Detection of anti-ganglioside autoantibodies does not necessarily indicate clinical disease, but these autoantibodies are in the hypothesized disease pathway for autoimmune peripheral neuropathy, which is illustrated in Fig 1, and are used as outcome biomarkers in the present study.

Only one previous study, to our knowledge, has examined biomarkers of both exposure to *C*. *jejuni* and of autoimmune outcomes in workers exposed to animals compared to unexposed referents. Price et al. [5] reported that levels of anti-*C*. *jejuni* antibodies were significantly higher, and IgG anti-ganglioside autoantibodies were increased, in 18 male poultry-house workers compared to 18 male referents, but the autoantibody analysis indicated only suggestive associations (p = 0.074), likely due to the small sample size. The present study utilizes a larger sample of AHS swine farmers from Iowa, some of whom also farmed chickens or cattle, and assesses serum anti-*C*. *jejuni* antibodies and anti-ganglioside autoantibodies compared with a reference group drawn from non-farmers.

In this study we tested the following hypotheses: (1) Farmers who work with animals will have higher levels of anti-*C*. *jejuni* antibodies compared to non-farmers. (2) Anti-*C*. *jejuni* antibody levels among farmers will vary based on animal herd or flock size. (3) Animal farmers will be more likely to test positive for anti-ganglioside autoantibodies compared to non-farmers. (4) Higher anti-*C*. *jejuni* antibody levels will be associated with an increased frequency of detection of anti-ganglioside autoantibodies.

## Materials and Methods

### Study Sample

This study analyzed serum samples and questionnaire data from a subset of participants in previous influenza studies that included AHS participants and controls. The AHS is an ongoing longitudinal study of 52,394 private pesticide applicators (mostly farmers), 32,345 spouses of the applicators, and 4,916 commercial pesticide applicators in Iowa and North Carolina. Phase I of the AHS, including an enrollment questionnaire and a take-home farmer applicator questionnaire, was conducted from 1993–1997 [26]. The present study accessed data from AHS Phase I Release P1REL201005.00, including information on animal exposures and neurologic symptoms.

For the studies of swine and avian influenza, Gray and colleagues collected serum samples during 2004–2006 from 803 AHS private pesticide applicators and spouses of applicators from Iowa who were selected based on exposure to swine or poultry [1,2]. These studies also included assessment of serum samples from 79 controls originally recruited for another study, who were generally healthy University of Iowa students, staff, and faculty who denied having swine or poultry exposure [1,2]. The recruitment methods for the influenza studies have been described previously [1,2]. The present study utilized questionnaire data and sera collected in 2006 from a sub-sample of the participants in these influenza studies.

**Fig 2:**
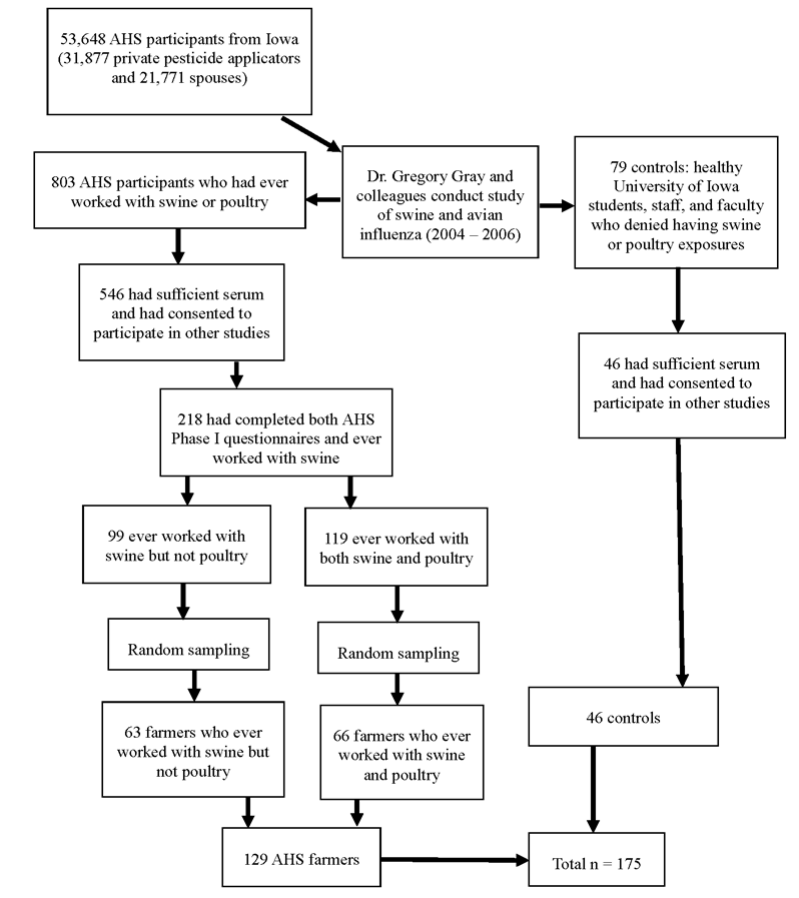
Study Population. AHS = Agricultural Health Study.

The selection of the study population is illustrated in Fig 2. Of the 803 AHS participants in the influenza studies, 546 were documented as having greater than one ml of serum remaining available and had consented to participate in other studies. Of these, 219 had completed both the enrollment and take-home questionnaires from AHS Phase I, including questions on five neurologic symptoms of interest. All but one of these participants reported ever working with swine. Of the 218 participants who had reported working with swine, 119 had also reported working with poultry. Random sampling was used to select 66 out of 119 participants who had ever worked with both swine and poultry and 63 out of 99 participants who had ever worked with swine but not poultry for participation in the current study. Out of the 79 controls in the influenza studies, 46 met the criteria of having a sufficient quantity of sera available and consenting to participate in other studies. Serum samples from all 46 of the available controls were analyzed in the present study.

The Gray laboratory at the University of Iowa sent 181 blinded serum samples to the Johns Hopkins Bloomberg School of Public Health (JHSPH) in two aliquots of 0.25 ml each. Six of these samples were duplicates that were used for quality control analyses, so the final study sample size was 175: 129 AHS participants and 46 controls. Of the 129 AHS participants, 126 were male and three were female. All of the male AHS participants were private pesticide applicators. Of the females, one was a private applicator and two were spouses of applicators. All of the AHS participants had reported working with animals at some point, and therefore were classified as farmers.

### Ethics Statement

Participants signed written informed consent forms to participate in the influenza studies, including consent for their data and sera to be used in future studies. The Institutional Review Board (IRB) of the Johns Hopkins Bloomberg School of Public Health determined that the present study (IRB # 00001706), which analyzes pre-existing data and de-identified serum samples that had been collected under the protocols of previous studies, does not qualify as human subjects research.

### Serum Analyses

One set of serum samples was sent to the Nachamkin laboratory at the University of Pennsylvania for anti-*C*. *jejuni* antibody analyses. Serum samples were analyzed for IgA, IgG, and IgM anti-*C*. *jejuni* antibodies using enzyme-linked immunosorbent assays (ELISA), as described previously [27–29]. The optical density (OD) for each sample was compared to the threshold value for sera run at the same time and obtained from a separate population of asymptomatic individuals, to get an optical density ratio (ODR) [27–29]. The ODR was treated as a continuous variable for data analysis. For the six individuals with duplicate samples, the average ODR of the two samples was used for analysis.

The other set of samples was sent to the Sheikh laboratory at the University of Texas for anti-ganglioside autoantibody analyses. Using ELISA, serum samples were tested for autoantibodies to five gangliosides: GM1, GD1a, GD1b, GQ1b, and SGPG, for the antibody classes of IgG and IgM, using methods that have been described previously [27,30]. Each sample was run on one or more ganglioside-coated wells and also on a blank well. The OD for the blank well was subtracted from the mean OD for the coated wells to get an adjusted OD. Previous research found that three standard deviations above the mean of blank wells gave an OD of 0.1 [30]. Therefore, samples were considered positive if the adjusted OD was greater than or equal to 0.1, as in previous studies [27,30]. For the six individuals with duplicate samples, if either of the two samples had an adjusted OD greater than or equal to 0.1, then the individual was considered positive for that autoantibody. Anti-ganglioside autoantibodies could not be assessed for two of the samples because of insufficient volume.

The difference between the two duplicate samples for the six individuals was not statistically significantly different than zero by Wilcoxon Signed-Rank Test for anti-*C*. *jejuni* IgA (p = 0.094), IgG (p = 0.69) or IgM (p = 0.69) antibodies. The difference in anti-ganglioside autoantibody adjusted OD between the duplicate samples was not statistically significantly different from zero (p > 0.06) for IgG anti-ganglioside autoantibodies, with the exception of IgG anti-GQ1b autoantibodies, for which the median difference between the two duplicate samples was 0.15 (p = 0.031). The difference in anti-ganglioside autoantibody adjusted OD between the duplicate samples was not statistically significantly different from zero (p > 0.06) for any IgM anti-ganglioside autoantibodies. When autoantibody detection was considered as dichotomous (positive or negative), two individuals were discordant on the results for their duplicate samples for IgG anti-SGPG autoantibodies and two other individuals were discordant on the results for their duplicate samples for IgM anti-SGPG autoantibodies.

### Questionnaire Data

Questionnaire data from the influenza studies were used to assess participant demographics and exposures. Animal exposure was based on the question “Please indicate if you have worked with these animals in the past year and the size of the herd/flock: Swine, Chickens, Cattle, Horses, Turkeys, Sheep, Other (please list).” Based on the responses to this question, we categorized participants as having worked with swine, chickens, cattle, other poultry, or other animals at the time of serum collection. Participants were classified as having worked with other poultry if they reported work with turkeys or listed any of the following as other: ducks, geese, or pea fowl.

We also classified animal exposure based on reported herd/flock size at the time of serum collection for swine, chickens, and cattle, into categories of zero, small, and large. The cutoff for small vs. large herd/flock size was based on the median number of animals for those with more than zero animals. For swine and cattle, we also did a sensitivity analysis using a definition of small vs. large herd that was based on the U.S. Environmental Protection Agency (EPA) definition of a medium swine concentrated animal feeding operation (CAFO), or a medium mature dairy cattle CAFO [31]. Herd/flock size categories were treated as dummy variables in regression modeling, with herd/flock size of zero as the reference group.

### Statistical Analyses

The distributions of anti-*C*. *jejuni* ODRs were log-normal and comparisons of anti-*C*. *jejuni* antibody levels between groups were analyzed using non-parametric Mann-Whitney (Wilcoxon Rank-Sum) Tests. Because only three of the AHS participants were female, adjustment for sex between farmers and controls was not performed. The three female AHS participants were included in comparisons to controls but were excluded from comparisons that were conducted only among AHS participants to reduce potential confounding. Regression modeling was used to compare log-transformed anti-*C*. *jejuni* antibody ODR levels by age. Stratification by age was performed for the comparison of anti-*C*. *jejuni* antibody levels between farmers and controls because the age distributions differed between groups. Regression modeling with dummy variables was used to compare log-transformed anti-*C*. *jejuni* antibody ODR distributions of the three categorical groups of herd/flock size for farmers. Comparisons of anti-ganglioside autoantibodies between groups were conducted using Fisher’s Exact Test, and exact logistic regression modeling was used to adjust for potential confounders. Logistic regression modeling was used to assess the association between log-transformed anti-*C*. *jejuni* antibody levels and positivity for anti-ganglioside autoantibodies. Comparison of laboratory results for the six duplicate samples was assessed using Wilcoxon Signed-Rank Tests. All statistical analyses were conducted using SAS® 9.2 software (SAS Institute, Cary, NC). Graphs of antibody distributions were produced using Stata 11.0 (Statacorp, College Station, TX) after conversion from SAS using StatTransfer.

## Results

### Demographics and animal exposures

The demographic characteristics of the two groups are presented in Table 1. The referent population was drawn from a previous study, which recruited students, faculty, and staff at the University of Iowa. As a consequence, there were significant differences between the two groups in terms of sex and age. Ninety-eight percent of the AHS farmers were male, whereas only 30% of controls were male. Farmers and controls also differed significantly in age distribution, as shown in Table 1, with farmers skewed towards older and controls skewed towards younger ages.

**Table 1:** Characteristics of All Study Participants (n = 175).

	University of Iowa Controls (n = 46)	AHS Farmers Who Ever Worked With Swine (n = 129)	Total (n = 175)
Male*	14 (30%)	126 (98%)	140 (80%)
**Smoker**	2 (4%)	11 (9%)	13 (7%)
**Age**			
**18–45**	26 (56%)	14 (11%)	40 (23%)
**46–55**	10 (22%)	30 (23%)	40 (23%)
**> 55**	10 (22%)	85 (66%)	95 (54%)
Mean Age (SD)**	42.0 (15.6)	60.1 (11.3)	55.3 (14.8)

*Controls and farmers differ in sex significantly by Fisher’s Exact Test.

**Mean age is significantly different between controls and farmers by t-test.

**Fig 3:**
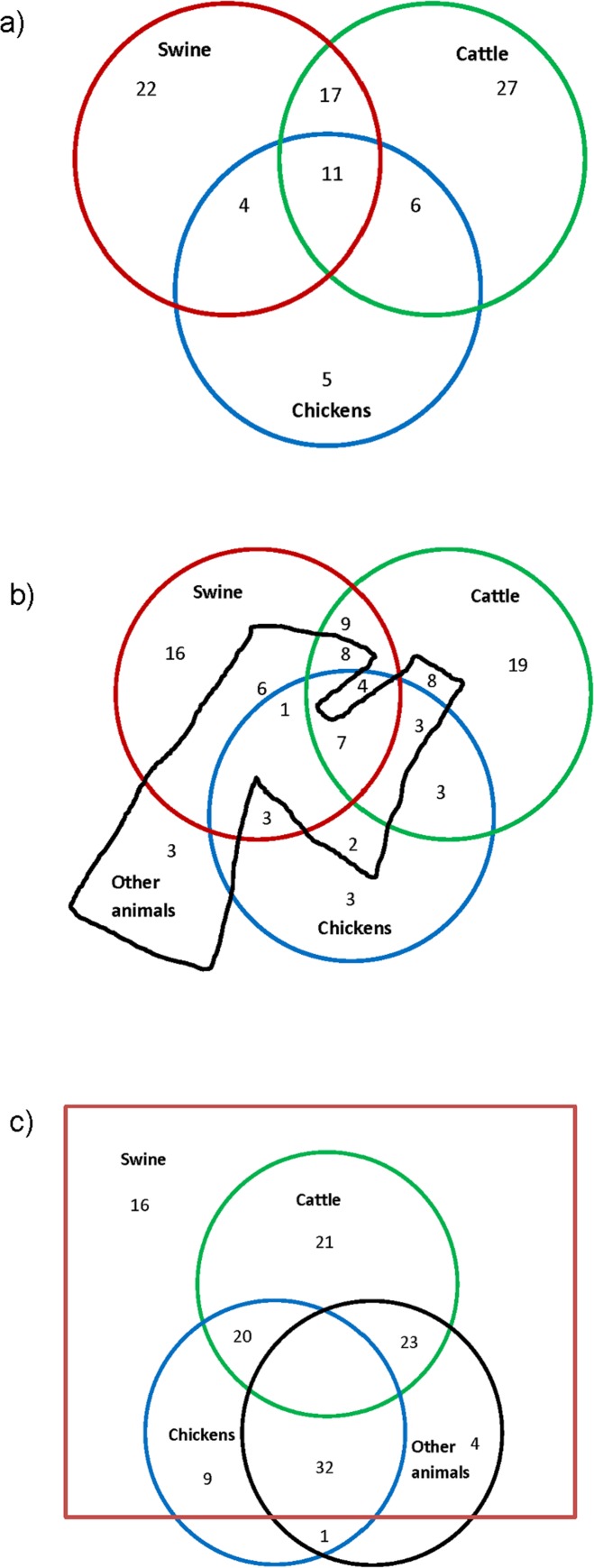
Venn Diagrams Illustrating the Challenges of Defining or Categorizing Exposures. **a) Numbers of Male Farmers Who Reported Working with Swine, Cattle, and Chickens in 2006.** A total of 95 out of 126 male farmers reported working with animals in 2006. This diagram includes 92 of these farmers and excludes three farmers who reported working only with other animals. A total of 54 male farmers reported working with swine, 61 reported working with cattle, and 26 reported working with chickens. The overlap between these categories is illustrated in the Fig Circles are not drawn to scale. **b) Numbers of Male Farmers Who Reported Working with Swine, Cattle, Chickens, and Other Animals in 2006.** A total of 95 out of 126 male farmers reported working with animals in 2006: 54 reported working with swine, 61 reported working with cattle, 26 reported working with chickens, and 38 reported working with other animals. The overlap between these categories is illustrated in the Fig, which is not drawn to scale. “Other animals” includes horses (n = 20), sheep (n = 17), poultry other than chickens (n = 6), goats (n = 6), and other animals (n = 6), with some farmers reporting working with more than one type of other animal. **c) Numbers of Male Farmers Who Reported Ever Working With Swine, Cattle, Chickens, and Other Animals.** This diagram includes all 126 male farmers who reported ever working with animals. Of these, 125 reported working with swine, 64 reported working with cattle, 62 reported working with chickens, and 60 reported working with other animals. The overlap between these categories is illustrated in the Fig, which is not drawn to scale. “Other animals” includes horses (n = 32), sheep (n = 38), poultry other than chickens (n = 12), goats (n = 6), and other animals (n = 11), with some farmers reporting working with more than one type of other animal.

Most (97%) of the AHS farmers in the present study reported working with at least one type of animal on the AHS enrollment questionnaire in 1993–1997. At the time of serum collection in 2006 (approximately 10 years after enrollment), 75% reported working with animals. All of the farmers were included in the study based on the criterion of having worked with swine at some point, but one farmer in the study was misclassified and had not reported working with swine. The numbers of male farmers who reported working with swine, chickens, and cattle in 2006 and the overlap between these categories is shown in Fig 3A. Fig 3B illustrates the numbers of farmers in these categories who also reported work with other animals. Fig 3C shows the overlap between the numbers of male farmers who reported ever working with swine, chickens, cattle, and other animals.

### Findings of Antibody Analyses

**Fig 4:**
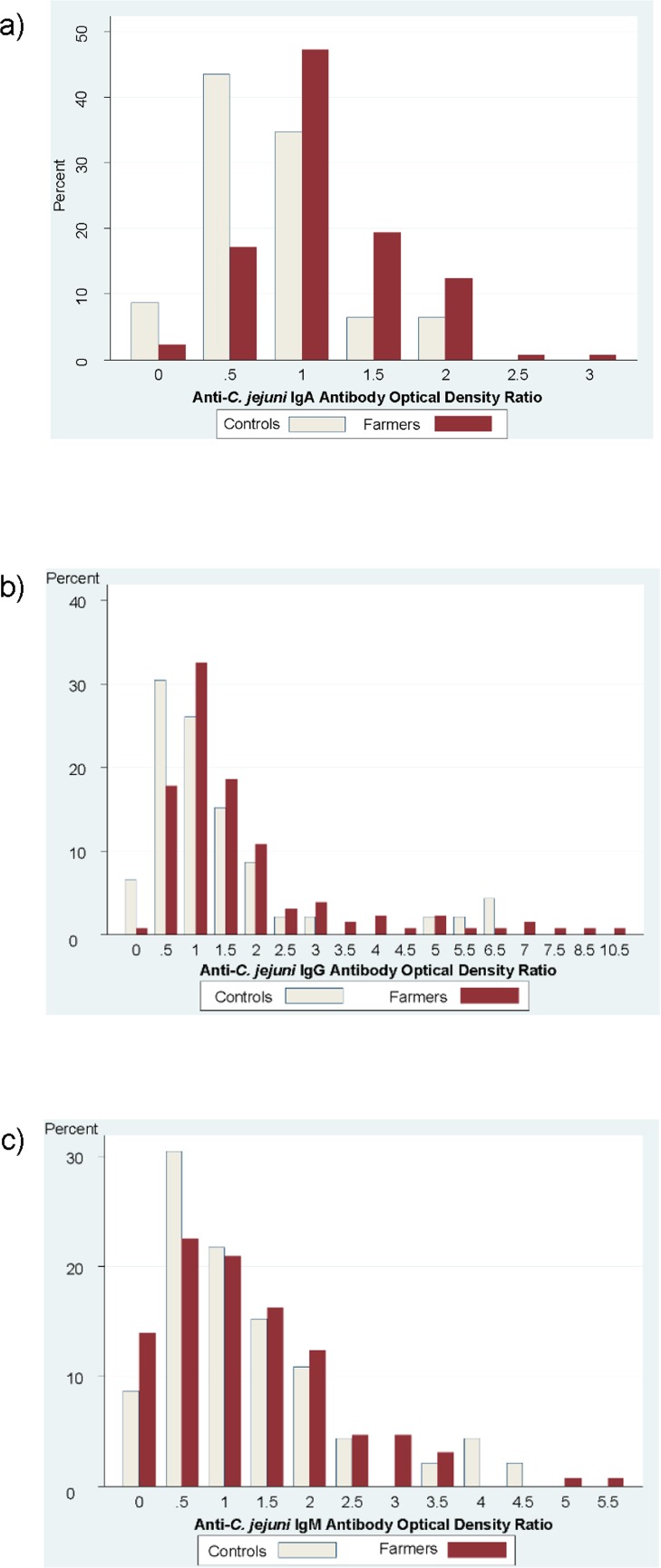
Anti-*C*. *jejuni* Antibody Optical Density Ratios for Farmers Compared to Controls: a) IgA; b) IgG; c) IgM.

The farmers had significantly higher levels of anti-*C*. *jejuni* IgA and IgG antibodies compared to the non-farmers (Table 2 and Fig 4A and 4B). Farmers and non-farmers did not significantly differ in levels of IgM anti-*C*. *jejuni* antibodies (Table 2 and Fig 4C).

**Table 2:** Anti-*Campylobacter jejuni* Antibodies: Farmers vs. Controls (n = 175).

Antibody Class	University of Iowa Controls (n = 46)	AHS Farmers Who Ever Worked With Swine (n = 129)	Wilcoxon Rank-Sum (Mann-Whitney) p-value
**IgA**			
**Median ODR (IQR)**	**0.99 (0.54)**	**1.34 (0.64)**	**p < 0.0001**
**Mean ODR (95% CI)** *	**0.97 (0.86–1.09)**	**1.29 (1.20–1.38)**	
**IgG**			
**Median ODR (IQR)**	**1.10 (0.99)**	**1.49 (1.06)**	**p = 0.023**
**Mean ODR (95% CI)** *	**1.30 (1.07–1.56)**	**1.62 (1.45–1.81)**	
**IgM**			
**Median ODR (IQR)**	1.21 (1.17)	1.24 (1.26)	p = 0.95
**Mean ODR (95% CI)** *	0.99 (0.65–1.51)	1.02 (0.79–1.31)	

Findings that are significant at p < 0.05 are bolded.

ODR = Optical Density Ratio. IQR = interquartile range. CI = confidence interval.

* Exponentiated mean and (95% confidence interval) of log-transformed anti-*C*. *jejuni* antibody ODR.

Anti-*C*. *jejuni* IgA antibody levels were higher at older ages in the whole sample (farmers and controls) (p = 0.0037). When controls were assessed separately, anti-*C*. *jejuni* IgA antibody levels were higher at older ages, but with borderline statistical significance (p = 0.066). Anti-*C*. *jejuni* IgA antibodies did not vary significantly by age in farmers (p = 0.76). Anti-*C*. *jejuni* IgG and IgM antibody levels did not vary significantly by age in the whole sample (p = 0.91 and p = 0.089, respectively) or when controls and farmers were analyzed separately (p > 0.10).

The differences in anti-*C*. *jejuni* IgA, IgG, and IgM ODR distributions between farmers and non-farmers stratified by age are presented in Table 3. For anti-*C*. *jejuni* IgA and IgG antibodies, farmers had statistically significantly higher ODRs compared to non-farmers in the 18–45 age category, borderline significantly higher ODRs in the 46–55 age category, and did not significantly differ in ODR distributions in the over 55 age category. There was no statistically significant difference in anti-*C*. *jejuni* IgM ODR distributions between farmers and controls for any of the three age categories.

**Table 3:** Difference in Anti-*C*. *jejuni* Optical Density Ratio Distribution Between Farmers and Controls by Age Category (n = 175).

Age Group (Years)	University of Iowa Controls (n = 46)	AHS Farmers (n = 129)	p-value for Difference
	**IgA Median ODR**	**IgA Median ODR**	
**18–45** ^**a**^	**0.98**	**1.22**	**p = 0.02**
**46–55** ^**b**^	0.99	1.41	p = 0.08
**> 55** ^**c**^	1.20	1.29	p = 0.61
	**IgG Median ODR**	**IgG Median ODR**	
**18–45** ^**a**^	**1.00**	**2.06**	**p = 0.02**
**46–55** ^**b**^	1.11	1.69	p = 0.13
**> 55** ^**c**^	1.55	1.46	p = 0.53
	**IgM Median ODR**	**IgM Median ODR**	
**18–45** ^**a**^	1.33	1.47	p = 0.66
**46–55** ^**b**^	1.59	1.88	p = 0.54
**> 55** ^**c**^	0.83	1.15	p = 0.30

Findings that are significant at p < 0.05 are bolded.

^**a**^ n = 26 controls and 14 farmers.

^**b**^ n = 10 controls and 30 farmers.

^**c**^ n = 10 controls and 85 farmers.

Male farmers with no reported animals at the time of serum collection (n = 31) did not differ significantly in anti-*C*. *jejuni* IgA (p = 0.79) or IgM (p = 0.89) antibody levels from those who reported one or more animals at the time of serum collection (n = 95). Male farmers who reported one or more animals at the time of serum collection had higher (p = 0.001) anti-*C*. *jejuni* IgG ODRs (median 1.70, IQR 1.36) compared to male farmers who reported zero animals at the time of serum collection (median 1.24, IQR 0.59).

The anti-*C*. *jejuni* antibody ODR comparisons by animal herd or flock size at the time of serum collection for male farmers are presented in Table 4. Anti-*C*. *jejuni* antibodies did not vary significantly by swine herd size, except that male farmers with large swine herd sizes had lower levels of anti-*C*. *jejuni* IgM antibodies compared to male farmers with zero swine at the time of serum collection, and this was of borderline statistical significance (p = 0.053). Results were similar when swine herd size was categorized using the CAFO-based definition. Male farmers with small chicken flocks had significantly lower levels of anti-*C*. *jejuni* IgA antibodies (p = 0.017) and borderline significantly lower levels of anti-*C*. *jejuni* IgM antibodies (p = 0.094) than male chicken farmers with zero chickens at the time of serum collection. Male farmers with small cattle herds had higher levels of anti-*C*. *jejuni* IgG antibodies than male farmers with zero cattle at the time of serum collection (p = 0.001), as did male farmers with large cattle herds (p = 0.005). Results followed a similar trend when cattle herd size was categorized using the CAFO-based definition. Results for animal herd or flock size were similar when adjusted for age.

**Table 4:** Anti-*C*. *jejuni* Antibody Optical Density Ratios (ODR) by Animal Herd or Flock Size in 2005–2006 for Male AHS Farmers.

Herd or Flock Size Category	Difference from Reference Group in log Anti-*C*. *jejuni* ODR (95% CI)	Exponentiated Difference from Reference Group in Anti-*C*. *jejuni* ODR (95% CI)	p-value
Swine Herd Size	**IgA**		
**Zero Swine (n = 72)**	Reference Group		
**1–500 Swine (n = 28)**	-0.09 (-0.27–0.09)	0.91 (0.77–1.09)	p = 0.30
**>500 Swine (n = 26)**	-0.04 (-0.22–0.15)	0.96 (0.80–1.16)	p = 0.70
**IgG**			
**Zero Swine (n = 72)**	Reference Group		
**1–500 Swine (n = 28)**	0.12 (-0.17–0.40)	1.12 (0.84–1.50)	p = 0.43
**>500 Swine (n = 26)**	0.06 (-0.23–0.36)	1.06 (0.79–1.43)	p = 0.68
**IgM**			
**Zero Swine (n = 72)**	Reference Group		
**1–500 Swine (n = 28)**	0.15 (-0.46–0.77)	1.17 (0.63–2.16)	p = 0.62
**>500 Swine (n = 26)**	-0.62 (-1.25–0.01)	0.54 (0.29–1.01)	p = 0.053
Chicken Flock Size	**IgA**		
**Zero Chickens (n = 100)**	Reference Group		
**1–50 Chickens (n = 15)**	**-0.27 (-0.49 –-0.05)**	**0.77 (0.62–0.95)**	**p = 0.02**
**>50 Chickens (n = 11)**	0.06 (-0.19–0.31)	1.06 (0.83–1.37)	p = 0.63
**IgG**			
**Zero Chickens (n = 100)**	Reference Group		
**1–50 Chickens (n = 15)**	0.07 (-0.28–0.43)	1.07 (0.75–1.53)	p = 0.69
**>50 Chickens (n = 11)**	0.22 (-0.19–0.63)	1.25 (0.83–1.88)	p = 0.28
**IgM**			
**Zero Chickens (n = 100)**	Reference Group		
**1–50 Chickens (n = 15)**	-0.65 (-1.42–0.11)	0.52 (0.24–1.12)	p = 0.09
**>50 Chickens (n = 11)**	0.52 (-0.36–1.40)	1.68 (0.70–4.03)	p = 0.25
Cattle Herd Size	**IgA**		
**Zero Cattle (n = 65)**	Reference Group		
**1–80 Cattle (n = 31)**	0.002 (-0.17–0.18)	1.00 (0.84–1.19)	p = 0.98
**>80 Cattle (n = 30)**	0.12 (-0.06–0.30)	1.13 (0.94–1.35)	p = 0.18
**IgG**			
**Zero Cattle (n = 65)**	Reference Group		
**1–80 Cattle (n = 31)**	**0.44 (0.17–0.71)**	**1.56 (1.19–2.03)**	**p = 0.001**
**>80 Cattle (n = 30)**	**0.39 (0.12–0.67)**	**1.47 (1.12–1.93)**	**p = 0.005**
**IgM**			
**Zero Cattle (n = 65)**	Reference Group		
**1–80 Cattle (n = 31)**	0.43 (-0.18–1.04)	1.54 (0.84–2.82)	p = 0.16
**>80 Cattle (n = 30)**	-0.10 (-0.72–0.51)	0.90 (0.49–1.67)	p = 0.74

Findings that are significant at p < 0.05 are bolded.

The anti-ganglioside autoantibody results for farmers compared to controls are presented in Table 5. Results are presented for each of the ten autoantibodies (five anti-ganglioside autoantibodies in two classes), as well as combined across antibody type and class, and overall positivity for any anti-ganglioside autoantibody. Most participants tested negative for any anti-ganglioside autoantibody. Four non-farmers (nine percent) and 27 farmers (21%) tested positive for at least one anti-ganglioside autoantibody. This difference was not statistically significant at the p < 0.05 level on Fisher’s Exact Test (odds ratio = 2.65, p = 0.11) or in logistic regression adjusted for age (odds ratio = 3.16, p = 0.10). Results for analyses of all swine farmers were similar for the subset of swine farmers who had ever worked with poultry.

**Table 5:** Anti-ganglioside Autoantibodies: Farmers vs. Controls (n = 173)*.

Number Positive for Each Type of Anti-ganglioside Autoantibody	University of Iowa Controls (n = 44)*	AHS Farmers Who Ever Worked With Swine (n = 129)	Unadjusted Odds Ratio (95% Confidence Interval)	Unadjusted Fisher’s Exact Test p-value	Exact Logistic Regression Adjusted for Age; Odds Ratio (95% Confidence Interval)	Exact Logistic Regression Adjusted for Age; p-value
**IgG GM1**	0 (0%)	4 (3%)	---	p = 0.57	1.37 (0.14 – ∞)	p = 0.81
**IgG GD1a**	0	0	---	---	---	---
**IgG GD1b**	0 (0%)	1 (1%)	---	p = 1.00	---	---
**IgG GQ1b**	0	0	---	---	---	---
**IgG SGPG**	2 (5%)	8 (6%)	1.39 (0.26–13.9)	p = 1.00	2.42 (0.31–33.9)	p = 0.62
**IgM GM1**	1 (2%)	6 (5%)	2.10 (0.24–98.7)	p = 0.68	1.03 (0.10–54.2)	p = 1.00
**IgM GD1a**	0 (0%)	1 (1%)	---	p = 1.00	---	---
**IgM GD1b**	0 (0%)	1 (1%)	---	p = 1.00	2.00 (0.05 – ∞)	p = 0.67
**IgM GQ1b**	0 (0%)	1 (1%)	---	p = 1.00	---	---
**IgM SGPG**	1 (2%)	8 (6%)	2.84 (0.36–129.0)	p = 0.45	4.05 (0.36–237.6)	p = 0.44
**All IgG**	2 (5%)	12 (9%)	2.15 (0.45–20.5)	p = 0.52	2.97 (0.46–35.9)	p = 0.38
**All IgM**	2 (5%)	15 (12%)	2.76 (0.60–25.8)	p = 0.24	2.69 (0.47–29.8)	p = 0.40
**GM1**	1 (2%)	10 (8%)	3.61 (0.49–160.4)	p = 0.29	2.23 (0.25–111.3)	p = 0.83
**GD1a**	0 (0%)	1 (1%)	---	p = 1.00	---	---
**GD1b**	0 (0%)	2 (2%)	---	p = 1.00	0.63 (0.04 – ∞)	p = 1.00
**GQ1b**	0 (0%)	1 (1%)	---	p = 1.00	---	---
**SGPG**	3 (7%)	16 (12%)	1.94 (0.51–10.9)	p = 0.41	3.35 (0.67–24.4)	p = 0.18
**Any anti-ganglioside**	4 (9%)	27 (21%)	2.65 (0.84–11.0)	p = 0.11	3.16 (0.83–15.7)	p = 0.10

*Antiganglioside autoantibody data were missing for two participants (controls), so they were excluded from these analyses.

There was no statistically significant association between anti-*C*. *jejuni* IgA (p = 0.99), IgG (p = 0.76), or IgM (p = 0.48) antibody levels and positivity for anti-ganglioside autoantibodies in male farmers, or in all participants (farmers and controls) for IgA (p = 0.14), IgG (p = 0.87), or IgM (p = 0.43) anti-*C*. *jejuni* antibodies.

## Discussion

This study investigated the likelihood that biomarkers separately related to exposure and response among farmers exposed to swine, poultry, and cattle would be elevated compared to a convenience sample of referents without known farm exposures. Farmers with past or present animal exposure had higher serum levels of IgA and IgG anti-*C*. *jejuni* antibodies than non-farmers, supporting the hypothesis that working with farm animals is associated with increased exposure to *C*. *jejuni*. The finding that farm animal exposure is associated with increased levels of anti-*C*. *jejuni* antibodies adds to the evidence that *C*. *jejuni* exposure is an occupational health hazard in the farm environment that should be addressed. Farmers also were more likely to test positive for anti-ganglioside autoantibodies, a trend in the direction of our hypothesis that occupational exposure to farm animals is associated with biomarkers of autoimmune peripheral neuropathy, but this finding did not reach statistical significance.

Previous studies of long-term *Campylobacter* exposure have found that anti-*C*. *jejuni* IgG antibody levels can remain high for at least two years after infection, whereas anti-*C*. *jejuni* IgA and IgM antibody levels increase shortly after infection and then decrease rapidly over the next few months [32–35]. For example, a two-year longitudinal study of 210 people with symptomatic stool-culture confirmed *Campylobacter* infection found that among anti-*Campylobacter* antibodies, IgG were the most variable between individuals (consistent with our findings in Fig 4), and remained at high levels in some individuals throughout the two-year follow-up period; IgM antibodies were generally elevated in the two months following infection and then decreased; and the IgA response declined steeply in the two months after infection and then remained low in most individuals [33].

Studies of exposure to poultry and other farm animals are consistent with this pattern. Price et al. [5] found that poultry-house workers had increased levels of anti-*C*. *jejuni* IgG, but not IgA, antibodies compared to community referents. An earlier study found that dairy farmers in Colorado had higher anti-*C*.*jejuni* IgG antibody titers than healthy hospital personnel, but that these two groups did not significantly differ in anti-*C*. *jejuni* IgM titers [32]. The dairy farmers in that population may have been exposed to *C*. *jejuni* either by contact with cattle or by consumption of raw milk, but both would generally be chronic rather than acute exposures [32]. Chronic exposure to raw milk has been associated with an increase in anti-*C*. *jejuni* IgG antibodies [29]. Long-term poultry slaughterhouse workers in Sweden were reported to have higher anti-*C*. *jejuni* IgG antibody levels compared to short-term slaughterhouse workers and blood donors [19]. An earlier study found that poultry slaughterhouse workers in Sweden had higher levels of both anti-*C*. *jejuni* IgG and IgM antibodies compared to samples of blood donors and pregnant women [36]. IgM antibodies generally are associated with an earlier stage of immune response, which is consistent with our findings that farmers with long-term exposures to animals did not differ from controls in levels of anti-*C*. *jejuni* IgM antibodies. Our finding of increased anti-*C*. *jejuni* IgA antibodies in the farmers, however, suggests that IgA antibody levels may also remain elevated under some circumstances. The IgA findings might have been influenced by the reliability and accuracy of the laboratory results. When we compared the six duplicate samples, there was some indication, though not statistically significant (p = 0.09), of a difference in anti-*C*. *jejuni* IgA findings between duplicate samples from individuals.

Our study found few differences among farmers in anti-*C*. *jejuni* antibody levels based on herd or flock size of animals at the time of serum collection. Differences that were observed were not consistent among swine, chicken, and cattle. The finding of a lack of a consistent association between herd or flock size and antibody levels might be because herd or flock size may not accurately represent the intensity of animal contact by farmers.

All except one of the farmers reported working with swine at some point in their lives, but by 2006, 72 out of 126 male farmers no longer had any swine. It is possible that farmers who developed symptoms of illness when working with animals may have eventually reduced or discontinued their work with these animals. This healthy worker effect might explain the lack of difference in anti-*C*. *jejuni* antibody levels between older farmers and controls. However, the farmers in this study also may have decreased farming as they got older or they may have stopped raising animals, consistent with national and statewide trends of consolidation in agriculture. USDA Census of Agriculture data show that the number of hog and pig farms in Iowa decreased from 17,585 farms in 1997 to 8,330 farms in 2007 [37]. During the past few decades, the farming of swine and other animals has shifted from small farms to larger industrial farms; many small independent farmers have left the farming profession [38]. There are several reasons, including paramount economic reasons, for these trends in exits from farming. For example, one study found that variables that were likely to influence an exit from dairy farming included older age, higher off-farm income, lower returns over variable costs, and greater diversification of farm income [39]. Thus, farmers in the present study may have stopped working with animals for economic reasons.

We hypothesized that farmers who worked with swine, poultry, or cattle would be more likely to test positive for any anti-ganglioside autoantibody than people who did not work with animals. The observed trend, although not statistically significant, was in this direction for all of the anti-ganglioside autoantibodies tested. It is possible that this finding may be due to chance, but Price et al. [5] similarly found that poultry-house workers were more likely to test positive for IgG anti-ganglioside autoantibodies, when the five autoantibodies that we assessed were considered as one group, but this result also was not significant at the p < 0.05 level (p = 0.074). Only a small proportion of people infected with *C*. *jejuni* develop clinically significant symptoms of GBS or other autoimmune peripheral neuropathies. The incidence of GBS after *C*. *jejuni* infection has been estimated to be about 1.17 per 1000 person-years based on a cohort study in the United Kingdom [40]. Characteristics of the host as well as of the bacteria play a role in immune response, including whether autoantibody proliferation and other features of autoimmune peripheral neuropathy occur [41]. *C*. *jejuni* bacteria with specific genetic characteristics and specific lipo-oligosaccharide structures are more strongly associated with cross-reactive antibodies and autoimmune peripheral neuropathy than other types of *C*. *jejuni* [42]. These more hazardous types of *C*. *jejuni* have been found at relatively high prevalence levels in U.S. poultry products [43], but the prevalence of different types of *C*. *jejuni* in the current study is unknown. Molecular analyses of *C*. *jejuni* could not be conducted in the current study because only human serum samples were available. Serum samples can provide information on antibody responses to pathogen exposures, but stool samples from currently infected individuals would be required to isolate *C*. *jejuni* bacteria. Although we tested for some of the more common anti-ganglioside autoantibodies, there are several other anti-ganglioside autoantibodies (such as GT1a, GD3, GM2, GD2, GA1, and GM1b) that are associated with autoimmune peripheral neuropathy, and it is possible that some of our study participants might have been positive for these other autoantibodies. In previous studies of *C*. *jejuni*-associated GBS, anti-ganglioside autoantibodies were not detected in some GBS patients [44]. Thus, the findings on anti-ganglioside autoantibodies are in the direction that we expected, but a limitation of the present study is the small sample size, and further study with a larger or more homogeneous sample would be needed to confirm the association. Because few studies have compared anti-ganglioside autoantibodies between workers exposed to animals and people who do not work with animals, it is difficult to estimate the expected proportions to use in a power or sample size calculation, but based on our study, we estimate that a total sample size of at least 270 would be needed to assess whether the trend that we observed is a true association.

The reliability and accuracy of the laboratory test results for anti-ganglioside autoantibodies, as well as the cutoff point for defining positive vs. negative results, might also have influenced the power to detect differences in these antibodies between farmers and controls. The finding for anti-SGPG autoantibodies of discordant (one positive and one negative) results for some duplicate samples is notable because anti-SGPG was the most commonly detected anti-ganglioside autoantibody and the discordance between duplicates indicates that if the assay had been repeated for more individuals, additional individuals might have been classified as positive for anti-SGPG autoantibodies. Future studies should include duplicate or triplicate antibody analyses if sufficient funding and other resources are available.

This study found no significant association between levels of anti-*C*. *jejuni* antibodies and the detection of anti-ganglioside autoantibodies. This finding is somewhat surprising, given the hypothesized model of disease progression presented in Fig 1, which is based on studies demonstrating molecular mimicry between *C*. *jejuni* and epitopes of human gangliosides [20–22]. However, this study was cross-sectional, and it is possible that the individuals with detectable anti-ganglioside autoantibodies had higher levels of anti-*C*. *jejuni* antibodies in the past. The ability to detect an association may also have been limited by the low prevalence of anti-ganglioside autoantibodies in the participants. In addition, although it is the most commonly identified antecedent to GBS, *C*. *jejuni* infection is not the only possible cause of autoimmune peripheral neuropathy, so the detected anti-ganglioside autoantibodies might be present due to other causes, including other pathogens [10].

In our previous studies of neurologic symptoms, we compared self-reported symptoms in farmers who reported working with poultry, swine, or cattle in AHS Phase I to self-reported symptoms in farmers who did not report work with animals [6,7]. Because only four AHS participants in the present study did not report working with animals during AHS Phase I (1993–1997), when data on neurologic symptoms were collected, this same analysis could not be performed. We did not have information on symptoms at the time of serum collection, and therefore could not assess symptoms as the endpoint outcome illustrated in Fig 1. Future antibody studies would benefit from the inclusion of questionnaire data on neurologic symptoms, and especially from the inclusion of clinical and diagnostic testing for GBS and other autoimmune peripheral neuropathies.

Our sample of farmers was relatively diverse in terms of potential exposures to animals, including various combinations of types of animals farmed (illustrated in Fig 3), which might have affected the antibody findings. All except one of the farmers had ever worked with swine, about half of them had also worked with poultry, 76% had worked with cattle, and some had worked with other animals. Many of the farmers had worked with animals in the past, but not during the year before serum collection. We attempted to assess time spent in the poultry house or swine barn, but did not have sufficient data to adequately quantify duration of exposure for many of the participants. Because the analyses used questionnaire data that had been obtained for a different purpose, the study was limited by a lack of information about whether the animals were housed indoors or outdoors, in confinement or free-roaming, or other animal housing conditions that might have contributed to differing exposures to pathogens including *C*. *jejuni*. Differences in job tasks may also result in differing exposures. A case-series of 29 laboratory-confirmed cases of *Campylobacter* (23 of which were *C*. *jejuni*) infection in a Virginia poultry slaughter and processing plant reported that 83% of the infected workers had worked at the plant for less than a month, 93% worked in first-processing areas including live-hang and evisceration, and 90% were residents of a temporary residential facility operated by the Virginia Department of Corrections [45]. Future studies with more detailed exposure assessments (including job tasks, type of animal housing, time spent in animal confinement areas, previous campylobacteriosis illness or diagnosis, drinking water source, and other potential exposure sources such as unpasteurized milk) would be beneficial to characterizing risk.

The study was limited by differences between the farmers and the control group, particularly in age and sex. Immune responses generally vary with age. People over 65 tend to have decreased antibody production and decreased switching of B-cell surface Ig from IgM to other classes [46]. A study conducted in England found that anti-*C*. *jejuni* IgG, but not IgA, antibody levels, were consistently higher at older ages [34]. In contrast, our results showed a significant increase in anti-*C*. *jejuni* IgA, but not IgG, antibody levels with increasing age. The lack of a significant association between anti-*C*. *jejuni* IgG antibody levels and age in our sample provides some evidence that the observed differences in anti-*C*. *jejuni* IgG antibody levels between farmers and controls were probably not due to age. Because of the possibility that the observed differences in anti-*C*. *jejuni* antibody levels, particularly IgA levels, between farmers and controls might be due to age, we also conducted analyses stratified by age, and found that significantly higher levels of anti-*C*. *jejuni* IgA and IgG antibodies in farmers compared to controls persisted in the youngest age group, but not in the oldest age group (Table 3). However, with only ten participants over age 55 in the control group, it is difficult to draw firm conclusions about the role of age in the anti-*C*. *jejuni* antibody findings. The anti-ganglioside autoantibody results did not change substantially when adjusted for age (Table 5). There was a slight increase in the odds ratio and decrease in the p-value for the association between animal farming and testing positive for any anti-ganglioside autoantibody when adjusted for age.

Because almost all of the farmers in the study were male, the role of sex in the findings is difficult to determine. We found that male farmers who reported one or more animals at the time of serum collection had higher anti-*C*. *jejuni* IgG ODRs compared to male farmers who reported zero animals at the time of serum collection, which provides some evidence that the observed differences between farmers and controls in anti-*C*. *jejuni* antibody levels are not likely to be entirely attributable to differences in sex between the two groups. However, immune responses are known to differ between males and females, with women having increased antibody production after infection [47]. Most autoimmune diseases have a higher incidence in women than in men, but the opposite is true for GBS [48]. A meta-analysis of studies conducted in North America and Europe estimated a relative risk of GBS for males compared to females of 1.78 (95% CI 1.36–2.33) [49]. We cannot rule out the possibility that observed differences in anti-ganglioside autoantibody positivity might be due to the differences in sex distribution of farmers and controls. A control group of farmers who did not work with animals would have reduced the potential for confounding, but was not available for this study.

The study has several strengths, including the use of previously stored serum samples and the use of biomarkers to assess exposure to *C*. *jejuni* and response as immune outcomes. The study findings add to the evidence that exposure to *C*. *jejuni* bacteria is an important occupational hazard for farmers who work with animals. Future studies with a larger, more homogeneous sample observed longitudinally would help to clarify the autoimmune and neurologic risks of these exposures. Efforts to reduce *C*. *jejuni* levels in poultry, swine, and cattle would benefit farmers, other food production workers, consumers, and the general public.
